# A Potent and Protective Human Neutralizing Antibody Against SARS-CoV-2 Variants

**DOI:** 10.3389/fimmu.2021.766821

**Published:** 2021-12-13

**Authors:** Sisi Shan, Chee Keng Mok, Shuyuan Zhang, Jun Lan, Jizhou Li, Ziqing Yang, Ruoke Wang, Lin Cheng, Mengqi Fang, Zhen Qin Aw, Jinfang Yu, Qi Zhang, Xuanling Shi, Tong Zhang, Zheng Zhang, Jianbin Wang, Xinquan Wang, Justin Jang Hann Chu, Linqi Zhang

**Affiliations:** ^1^ NexVac Research Center, Comprehensive AIDS Research Center, Center for Infectious Disease Research, Beijing Advanced Innovation Center for Structural Biology, School of Medicine, Tsinghua University, Beijing, China; ^2^ Biosafety Level 3 Core Facility, Yong Loo Lin School of Medicine, National University of Singapore, Singapore, Singapore; ^3^ Laboratory of Molecular RNA Virology and Antiviral Strategies, Department of Microbiology and Immunology, Yong Loo Lin School of Medicine, National University of Singapore, Singapore, Singapore; ^4^ The Ministry of Education Key Laboratory of Protein Science, Beijing Advanced Innovation Center for Structural Biology, Beijing Frontier Research Center for Biological Structure, Collaborative Innovation Center for Biotherapy, School of Life Sciences, Tsinghua University, Beijing, China; ^5^ School of Life Sciences, Tsinghua-Peking Center for Life Sciences, Tsinghua University, Beijing, China; ^6^ Institute for Hepatology, National Clinical Research Center for Infectious Disease, Shenzhen Third People’s Hospital, The Second Affiliated Hospital, School of Medicine, Southern University of Science and Technology, Shenzhen, China; ^7^ Infectious Disease Translation Research Programme, Yong Loo Lin School of Medicine, National University of Singapore, Singapore, Singapore; ^8^ Beijing Youan Hospital, Capital Medical University, Beijing, China; ^9^ Institute of Biopharmaceutical and Health Engineering, Tsinghua Shenzhen International Graduate School, Tsinghua University, Shenzhen, China; ^10^ Institute of Biomedical Health Technology and Engineering, Shenzhen Bay Laboratory, Shenzhen, China

**Keywords:** SARS-CoV-2, variants of concern, human neutralizing antibody, *in vivo* protection, epitope

## Abstract

As severe acute respiratory syndrome coronavirus 2 (SARS-CoV-2) variants continue to emerge and spread around the world, antibodies and vaccines to confer broad and potent neutralizing activity are urgently needed. Through the isolation and characterization of monoclonal antibodies (mAbs) from individuals infected with SARS-CoV-2, we identified one antibody, P36-5D2, capable of neutralizing the major SARS-CoV-2 variants of concern. Crystal and electron cryo-microscopy (cryo-EM) structure analyses revealed that P36-5D2 targeted to a conserved epitope on the receptor-binding domain of the spike protein, withstanding the three key mutations—K417N, E484K, and N501Y—found in the variants that are responsible for escape from many potent neutralizing mAbs, including some already approved for emergency use authorization (EUA). A single intraperitoneal (IP) injection of P36-5D2 as a prophylactic treatment completely protected animals from challenge of infectious SARS-CoV-2 Alpha and Beta. Treated animals manifested normal body weight and were devoid of infection-associated death up to 14 days. A substantial decrease of the infectious virus in the lungs and brain, as well as reduced lung pathology, was found in these animals compared to the controls. Thus, P36-5D2 represents a new and desirable human antibody against the current and emerging SARS-CoV-2 variants.

## Introduction

As the severe acute respiratory syndrome coronavirus 2 (SARS-CoV-2) continues to rage around the world, multiple variants have emerged and spread rapidly. Currently, four major variants of concern (VOCs) have been designated: Alpha (B.1.1.7), initially identified in the UK; Beta (B.1.351) in South Africa; Gamma (P.1) in Brazil; and Delta (B.1.617.2) in India (1–7). These VOCs are not only rapidly displacing local SARS-CoV-2 variants but are also carrying mutations in the N-terminal domain (NTD) and receptor-binding domain (RBD) on the spike protein that are critical for interactions with the ACE2 receptor and neutralizing antibodies (8–21). Conserved among variants Alpha, Beta, and Gamma, the N501Y mutation was previously shown to enhance the binding affinity to ACE2 (22, 23). The Beta and Gamma variants each has three mutation sites in common within the RBD region—K417N/T, E484K, and N501Y—which change their antigenic profiles. The Delta variant contains the unique L452R and T478K mutations while sharing a common mutation with the Alpha and Gamma variants at P681 near the furin cleavage site. Various deletion mutants in the NTD have also been identified, such as 69-70del and Y144del in Alpha and 242-244del in Beta. Mutations in other spike regions were also identified, and some are located in or proximal to major S protein antigenic sites and, therefore, may adversely affect the antibody neutralization induced by natural infection or through vaccination (12–14, 24). Serum from individuals vaccinated with the Moderna or Pfizer mRNA vaccine demonstrated a nearly eightfold reduction in virus neutralization capability to the Beta variant and fivefold to the Gamma variant, while retaining similar neutralizing activity to the Alpha variant compared to the wild-type virus (11, 19). The chimpanzee adenovirus-based vaccine, ChAdOx1 nCoV-19, given to individuals as a two-dose regimen failed to protect against mild to moderate coronavirus disease 2019 (COVID-19) caused by the Beta variant (25).

We and others have previously reported the isolation and characterization of several hundred RBD-specific mAbs from individuals infected with SARS-CoV-2 (26–35), some of which have been approved for emergency use authorization (EUA) or under active clinical development. Through structure and functional characterization, the RBD-specific antibodies were classified into four major categories (8, 10): class I antibodies, which are encoded by the *VH3-53* gene segment with short CDRH3 loops, block ACE2 and bind only to “up” RBDs; class II neutralizing antibodies include ACE2-blocking neutralizing antibodies that bind both “up” and “down” RBDs and can contact adjacent RBDs; class III neutralizing antibodies bind outside the ACE2 site and recognize both “up” and “down” RBDs; and class IV neutralizing antibodies, which do not block ACE2 and bind only to “up” RBDs. Most class I mAbs were largely disrupted by the K417N/T mutation and class II mAbs by the E484K mutation. Class III and IV mAbs were relatively less affected by the escape mutants (12–14). Fortunately, by screening multiple mAbs from convalescent or vaccinated individuals, a few broad and potent neutralizing antibodies directed to RBD were identified to neutralize or to *in vivo* protect against selected VOCs (19, 20, 36–39). These neutralizing antibodies may contribute to the residual serum neutralizing activity against SARS-CoV-2 VOCs in these individuals.

Here, we report on the isolation and characterization of a broad, potent, and protective antibody, P36-5D2, isolated from a SARS-CoV-2 convalescent individual and capable of neutralizing the major VOCs such as the Alpha, Beta, and Gamma variants. Through crystal and electron cryo-microscopy (cryo-EM) structure analyses, P36-5D2 was found to target a conserved epitope on the RBD of the spike protein, bypassing the three key mutations (K417N, E484K, and N501Y) responsible for immune escape from many potent neutralizing mAbs (12–14). P36-5D2 also demonstrated impressive protection in a transgenic mouse model against infection with either the SARS-CoV-2 Alpha or Beta variant. Treated animals demonstrated a substantial decrease in infectious virus load in the lungs and brain and reduced lung pathology compared to the control animals. Thus, P36-5D2 serves as an important reference for the development of next-generation antibody therapies against SARS-CoV-2 infection.

## Results

### P36-5D2 Shows Broad and Potent Neutralizing Activity Against Pseudotyped and Infectious SARS-CoV-2

We used flow cytometry to isolate memory B cells that recognized the fluorescence-labeled SARS-CoV-2 spike trimer as previously described (28). A total of 70 mAbs were able to bind to the SARS-CoV-2 spike, eight of which demonstrated neutralizing ability against the SARS-CoV-2 pseudovirus and infectious virus (**Table 1**, **Supplementary Figures S1A, B**). Four mAbs—P36-1A3, P36-1B7, P36-5D2, and P74-6D2—showed stronger neutralizing activity, with respective IC_50_ values of 0.015, 0.025, 0.053, and 0.025 μg/ml against pseudovirus (**Table 1**, **Supplementary Figure S1A**). The remaining four—P33-1F1, P33-3C5, P36-1C5, and P36-8F2—had relatively weaker neutralizing potency, with IC_50_ values ranging from 0.037 to 39.6 μg/ml, and failed to reach 90% neutralizing activity even at the highest concentration of 50 μg/ml (**Table 1**, **Supplementary Figure S1A**). The control and representative mAbs from each class, REGN10933 (class I), CB6 (class I), BD368-2 (class II), and REGN10987 (class III), were RBD-specific and showed comparable neutralizing activity to that reported (26, 27, 32). Another control NTD-specific mAb, 4A8 (40), had an IC_50_ of 0.115 μg/ml, but failed to reach 90% neutralization. Comparable neutralizing potency was also found for the eight isolated mAbs against the infectious SARS-CoV-2 wild-type (WT) strain (**Table 1**, **Supplementary Figure S1B**).

**Table 1:** Binding capacity, neutralizing activity, and gene family analysis of eight monoclonal antibodies (mAbs) isolated from individuals infected with SARS-CoV-2 and five published representative mAbs.

mAb name	Pseudovirus (μg/ml)		Infectious virus (μg/ml)		Binding to RBD		Heavy chain			Kappa chain or Lambda chain		
	IC50	IC90	IC50	IC90	KD (nM)	Comepting w/ ACE2	IGHV	CDR3 lengh	SHM%	IGK(L)V	CDR3 lengh	SHM%
P33-1F1	39.679	>50	27.918	>50	n.a.	n.a.	3-13*01	14	1.37	K 1-39*01	10	0.70
P33-3C5	0.037	>50	0.151	>50	n.a.	n.a.	1-24*01	14	1.70	L 2-14*01	10	0.34
P36-1A3	0.015	0.101	0.029	0.320	2.33	+++	3-66*01	9	1.02	K 1-9*01	8	0.00
P36-1B7	0.025	0.305	0.096	1.388	7.37	+++	3-66*01	11	0.68	K 1-9*01	10	0.00
P36-1C5	0.142	>50	0.154	>50	n.a.	n.a.	3-7*01	23	1.69	L 3-1*01	9	0.36
P36-5D2	0.053	0.910	0.060	0.513	6.91	+	1-3*01	15	0.68	K 1-5*01	9	0.71
P36-8F2	23.487	>50	22.476	>50	n.a.	n.a.	3-13*01	22	0.34	K 1-39*01	10	0.00
P74-6D2	0.025	0.302	0.075	1.260	4.11	+++	1-18*01	10	1.37	K 1-33*01	8	0.36
REGN10933(class I)	0.006	0.046	n.a.	n.a.	<0.01	+++	3-11*01	13	n.a.	K 1-33*01	9	n.a.
CB6(class I)	0.015	0.136	n.a.	n.a.	0.26	+++	3-66*01	13	n.a.	K 1-39*01	11	n.a.
BD368-2(class II)	0.004	0.023	n.a.	n.a.	0.07	+++	3-23*01	18	n.a.	K 2-28*01	9	n.a.
REGN10987(class III)	0.006	0.046	n.a.	n.a.	<0.01	+++	3-30*01	13	n.a.	L 2-14*03	10	n.a.
4A8(NTD)	0.115	>50	n.a.	n.a.	n.a.	n.a.	1-24*01	21	n.a.	K 2-24*01	9	n.a.

IC50 represents the half-maximal concentration and IC90 the 90% inhibitory concentration in the pseudovirus and infectious SARS-CoV-2 neutralization assay. Antibody binding to RBD was presented either by K_D_ or by competing with ACE2, in which +++ indicates >70% competition, ++ means 50%–70%, + denotes 20%–50%, and − indicates <20% competition. The program IMGT/V-QUEST was applied to analyze the gene germline, CDR3 length, and somatic hypermutation (SHM). The CDR3 length was calculated from the amino acid sequences.

The SHM frequency was calculated from the mutated nucleotides.

RBD, receptor-binding domain; IGHV, immunoglobulin heavy-chain variable region gene; n.a. not available.

**Figure 1:**
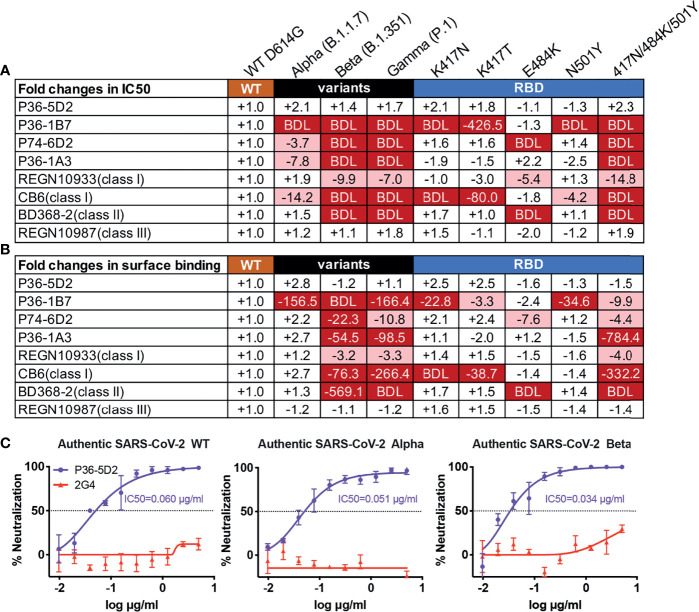
P36-5D2 shows broad and potent neutralizing and binding spectrum against pseudotyped and infectious SARS-CoV-2. P36-5D2, P36-1B7, P74-6D2, and P36-1A3 were the top 4 isolated neutralizing monoclonal antibodies (mAbs) against severe acute respiratory syndrome coronavirus 2 (SARS-CoV-2) from infected individuals. Published representative receptor-binding domain (RBD)-specific mAbs included REGN10933 (class I), CB6 (class I), BD356-2 (class II), and REGN10987 (class III). The neutralizing and binding ability of mAbs against pseudotyped SARS-CoV-2, including Alpha (B.1.1.7), Beta (B.1.351), and Gamma (P.1), and the respective mutations derived from the three variants of concern (VOCs) were evaluated. Values indicate the fold changes in half-maximal inhibitory concentrations (IC_50_) **(A)** and the mean fluorescence intensity (MFI) relative to that of wild-type (WT) D614G **(B)**. The IC_50_ of antibodies against WT D614G are listed in **(A)**. The *minus symbol* represents increased resistance and the *plus sign* indicates increased sensitivity. IC_50_ or MFI highlighted in *red* indicates that resistance increased at least threefold; in *blue*, sensitivity increased at least threefold. *BDL* (below detection limit) represents the highest concentration of mAbs that failed to reach 50% potency in neutralization activity or mAbs that failed to bind the cell surface-expressed SARS-CoV-2 variants. Results are presented as the mean value from three independent experiments. **(C)** Neutralization of P36-5D2 against the infectious SARS-CoV-2 WT, Alpha, and Beta variants. 2G4 is the negative control antibody specific for Ebola virus glycoprotein (EBOV GP). Experiments were performed in triplicate, and all data were presented as the means ± SEM.

To evaluate the neutralizing breadth of the top 4 neutralizing mAbs, we used the established panel of pseudoviruses including three of the most challenging SARS-CoV-2 VOCs, Alpha, Beta, and Gamma, as well as single mutants derived from the three VOCs (20). As shown in **Figure 1A**, **Supplementary Figure S2**, P36-5D2 was the most broad and potent against all the pseudotyped VOCs and variants tested, as was the class III control REGN10987. In contrast, P36-1B7, like the class I mAb CB6, was substantially impacted by the SARS-CoV-2 Alpha, Beta, and Gamma variants, largely due to K417N/T and N501Y mutations. P74-6D2 demonstrated reduced or loss of neutralizing activities to the three VOCs, which was largely attributed to E484K, similar to the class II control mAb BD368-2 and the class I control mAb REGN10933. P36-1A3 appeared to be heavily affected by N501Y and K417-E484K-N501Y mutations. Furthermore, the neutralizing patterns against these pseudotyped VOCs and variants correlated well with their binding avidity to cell surface-expressed VOCs and the mutant spike proteins (**Figure 1B**, **Supplementary Figure S3**). This result suggested that a compromised binding avidity is a major escape mechanism.

Subsequently, we evaluated the neutralizing ability of P36-5D2 against the infectious SARS-CoV-2 WT, Alpha, and Beta. Consistent with that from pseudoviruses, P36-5D2 showed broad neutralizing activity to all three infectious viruses, with IC_50_ values of 0.060, 0.051, and 0.034 μg/ml against the SARS-CoV-2 WT, Alpha, and Beta respectively (**Figure 1C**). The dissociation constant (*K*_D_) of P36-5D2 with the SARS-CoV-2 RBD was 6.91 nM, measured using surface plasmon resonance (SPR), which was similar to those of P36-1A3, P36-1B7, and P74-6D2 (**Supplementary Figure S1B**). Through competition analysis measured by SPR, we found that P36-5D2 partially competed while P36-1A3, P36-1B7, and P74-6D2 completely competed with ACE2 for binding to RBD (**Supplementary Figure S1C**). Competition among the four mAbs further showed that P36-5D2 recognized a less overlapping epitope, while the other three strongly competed among each other (**Supplementary Figures S1D, E**). These results indicated that P36-5D2 recognized a different epitope from those by the other three mAbs.

### P36-5D2 Binds to a Highly Conserved Epitope on RBD and Avoids Three Key Mutant Residues (K417N, E484K, and N501Y)

**Figure 2:**
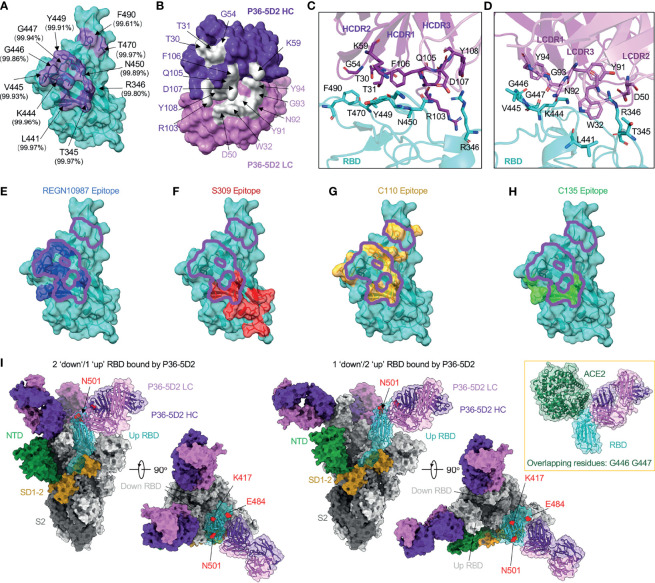
P36-5D2 binds to a highly conserved epitope on the receptor-binding domain (RBD) and avoids three key mutant residues: K417N, E484K, and N501Y. **(A)** The epitope of P36-5D2 (*purple*) is highlighted on the surface of severe acute respiratory syndrome coronavirus 2 (SARS-CoV-2) RBD-3M carrying K417T, E484K, and N501Y (*cyan*) (PDB: 7FJC). RBD-3M residues on the epitope are labeled. The percentages show the conservation at these positions observed in the GISAID EpiCoV database by aligning 1,959,333 sequences of the SARS-CoV-2 spike from December 2019 to July 2021. **(B)** The paratope of P36-5D2 (*sliver*) is highlighted on heavy chain (*purple*) and light chain (*pink*). Antibody residues are labeled in *purple* or *pink* depending on their origin from heavy or light chain. **(C, D)** Interaction between P36-5D2 Fab and SARS-CoV-2 RBD-3M. **(E–H)** Listed class III mAbs Fab fragments are superposed onto P36-5D2/RBD-3M crystal structure. REGN10987, S309, C110, and C135 are reference class III mAbs. The epitopes of these antibodies were assigned by selection of any RBD residue within 4 Å of any antibody residue. The epitope of REGN10987 (PDB: 6XDG) is colored in *blue*, S309 (PDB: 7JX3) in *red*, C110 (PDB: 7K8V) *yellow*, C135 (PDB: 7K8Z) *green*, and P36-5D2 in *purple*. **(I)** Electron cryo-microscopy (cryo-EM) for the P36-5D2 Fab and SARS-CoV-2 spike complex (*left*, 3.65 Å; *right*, 3.69 Å) reveals the binding of P36-5D2 Fab to both “up” and “down” RBDs. P36-5D2/RBD-3M crystal structure shown as cartoon superposed onto one protomer of P36-5D2/spike cryo-EM structure. The spike is shown as a molecular surface, with one protomer RBD colored *cyan*, NTD *green*, SD1-2 *yellow*, and S2 *gray*. The *top view* shows that P36-5D2 avoids residues K417, E484, and N501, which are shown as *red-colored spheres*. *Inset*: structure superposition showing clashes between ACE2 (PDB: 6MOJ; *green*) and P36-5D2 Fab. Overlapping residues shared between P36-5D2 and ACE2 are listed below.

To reveal the molecular basis of the broad and potent neutralizing activity of P36-5D2, we determined the crystal structure of its antigen-binding fragment (Fab) bound to the RBD-3M carrying the K417N, E484K, and N501Y mutations initially identified in the SARS-CoV-2 Beta variant. At a resolution of 3.1 Å, we found that P36-5D2 recognized an epitope consisting of 11 residues (T345, R346, L441, K444, V445, G446, G447, Y449, N450, T470, and F490) on RBD, devoid of the three key mutant residues K417N, E484K, and N501Y that facilitated escape from the neutralization of many mAbs, including some approved for EUA (**Figure 2A**). Over 99.5% conservation was found at all these sites among the 1.96 million spike sequences in the GISAID EpiCoV database collected from December 2019 to July 2021. The paratope of P36-5D2 consisted of nine heavy-chain residues—T30 and T31 of HCDR1; G54 and K59 of HCDR2; and R103, Q105, F106, D107, and Y108 of HCDR3—as well as six light-chain residues: W32 of LCDR1; D50 of LCDR2; and Y91, N92, G93, and Y94 of LCDR3 (**Figure 2B**). At the binding interface, R346 of the RBD had salt bridge interaction with D107 of HCDR3 and D50 of LCDR2, and K444, G446, and N450 had hydrogen bond interactions with residues Y91 of LCDR3 and K59, F106, and D107 of HCDR2 and HCDR3 (**Figures 2C, D** and **Supplementary Table S2**). Based on the binding specificity, P36-5D2 was closest to the class III RBD-targeting mAbs, including REGN10987, S309, C110, and C135 (8, 27, 30, 41). The P36-5D2 epitope overlapped more with that of REGN10987 and C110 than with S309 and C135 (**Figures 2E–H**). All these five class III mAbs shared two residues, R346 and L441, among their epitopes. Interestingly, P36-5D2, C110, and C135 had light-chain variable regions all derived from germline *IGKV 1-5*, indicating a selective preference for the particular germline gene during the development of this class of mAbs from different infected individuals.

We went further to determine the cryo-EM structures of the P36-5D2 Fab bound to the SARS-CoV-2 spike trimer at 3.7-Å resolution (**Figure 2I**). In one model, P36-5D2 Fabs bound two “up” RBDs and one “down” RBD. In the other model, P36-5D2 Fabs bound two “down” RBDs and one “up” RBD of the spike trimer. Consistent with the crystal structure analysis, the cryo-EM structure clearly demonstrated that the mutant residues K417N, E484K, and N501Y were not involved in the P36-5D2 epitope. Furthermore, P36-5D2 and ACE2 only had two overlapping residues at G446 and Y447, which was sufficient to exert spatial hindrance in disrupting the interaction between ACE2 and RBD. These results collectively revealed the highly conserved nature of the P36-5D2 epitope and provided a molecular basis for its broad and potent neutralizing activity against all the SARS-CoV-2 VOCs and mutant strains tested here.

**Figure 3:**
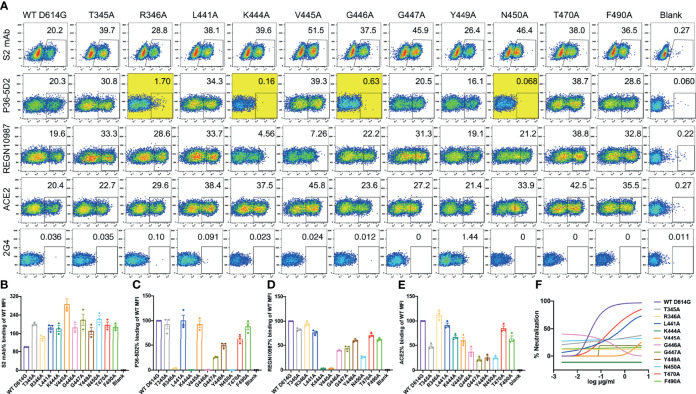
Impact of single alanine mutated residues on P36-5D2 binding and neutralization. **(A)** Wild-type and single alanine mutated spike were expressed on the surface of HEK 293T cells, incubated with P36-5D2, REGN10987, or ACE2, followed by staining with anti-human IgG Fc phycoerythrin (PE) or anti-His PE, and analyzed by FACS. For each panel, the *X*-axis means tested antibody or ACE2 binding PE/FITC and the *Y*-axis means side-scattered light (SSC). The gated cell percentages are shown. **(B)** The S2 monoclonal antibody (mAb) is a positive control antibody used for spike expression normalization. **(C–E)** The relative mean fluorescence intensity (MFI) of mAbs or ACE2 binding was determined by comparing the total MFI in the selected gate between the spike variants and WT D614G. 2G4 targeting Ebola virus glycoprotein (EBOV GP) is a negative control mAb. *NC* denotes HEK 293T cells with mock transfection. Alanine mutated residues completely destroying P36-5D2 binding are highlighted in *yellow*. Data are presented as the mean ± SEM from three independent experiments. **(F)** Impact of single alanine mutated residues on pseudovirus neutralization sensitivity to P36-5D2. Results are presented as the mean value from three independent experiments.

Next, we conducted a single-site alanine scanning mutagenesis for the 11 epitope residues within the P36-5D2 epitope to identify the key residues that mediate RBD binding. All 11 mutated spikes were successfully expressed on the surface of human embryonic kidney (HEK) 293T cells and can bind to the cellular receptor ACE2 (**Figures 3A, B, E**). Among the mutated residues, R346A, K444A, G446A, and N450A resulted in a near loss of P36-5D2 binding (**Figures 3A, C**). Two resides, K444A and V445A, also resulted in a substantial reduction in REGN10987 binding (**Figure 3D**), consistent with the epitope residues defined by structural analysis. Furthermore, among the 11 pesudoviruses bearing alanine mutated spikes, nine were able to confer resistance to P36-5D2, while the remaining two (L441A and T470A) had a relatively weaker impact (**Figure 3F**). These results suggested that the levels of binding to the surface-expressed spikes could not be accounted for during the entire neutralization of P36-5D2.

### P36-5D2 Protects K18-hACE2 Mice From Infectious SARS-CoV-2 Alpha or Beta Infection

**Figure 4:**
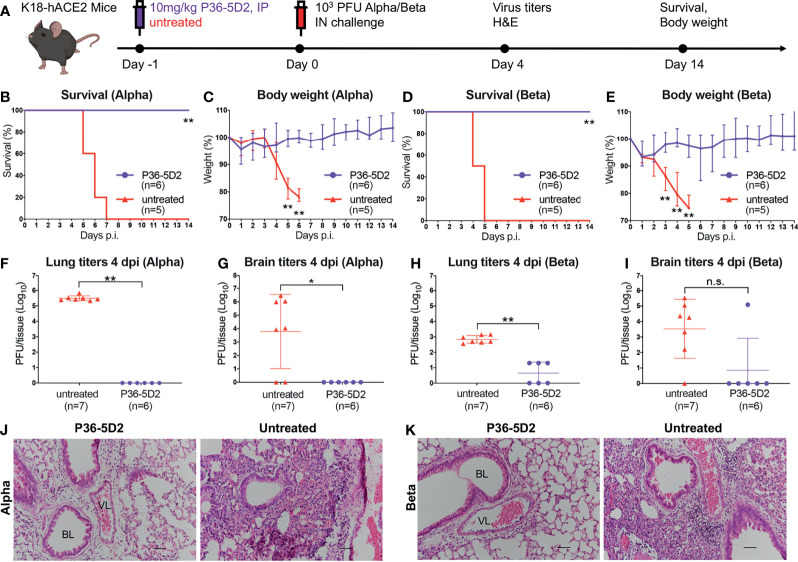
Efficacy of P36-5D2 prophylaxis against the infectious severe acute respiratory syndrome coronavirus 2 (SARS-CoV-2) Alpha or Beta variant in K18-hACE-2 mice. **(A)** Experimental schedule for antibody prophylaxis. Eight-week-old K18-hACE2 transgenic female mice were administered 10 mg/kg of P36-5D2 intraperitoneally or untreated 1 day prior to challenge with 10^3^ plaque-forming units (PFU) infectious SARS-CoV-2 Alpha or Beta *via* the intranasal route. **(B–E)** The survival percentage **(B, D)** and body weight **(C, E)** were recorded daily after infection until the occurrence of death or until the experimental end point at 14 days post-infection (dpi) (P36-5D2, *n* = 6; untreated, *n* = 5). Mice were sacrificed at 4 dpi for virus titer analysis (P36-5D2, *n* = 6; untreated, *n* = 7). **(F–I)** Lung titers **(F, H)** and brain titers **(G, I)** were tested by plaque assays in lung and brain tissue homogenates. The PFU per tissue were compared between groups in log_10_-transformed units. All data are presented as the mean ± SEM. Analysis of a two-tailed unpaired *t-*test was used. *p < 0.05, **p < 0.01. *n.s.*, not significant. **(J, K)** H&E staining of lung sections from P36-5D2-injected or untreated mice at 4 dpi. *VL*, vascular lumen; *BL*, bronchiolar lumen. *Scale bars*, 50 mm. Each image is representative of each group.

Thereafter, we evaluated the prophylactic potential of P36-5D2 against infectious SARS-CoV-2 Alpha or Beta infection in a well-established hACE2 transgenic mouse model (42, 43). The entire experimental protocol and assays conducted to evaluate the protection capability of P36-5D2 are outlined in **Figure 4A**. Briefly, 12 K18-hACE2 mice were IP administered with P36-5D2 at a dose of 10 mg/kg body weight a day before the intranasal (IN) challenge with 10^3^ plaque-forming units (PFU) SARS-CoV-2 Alpha or Beta variant. The mice were monitored for 14 days to record their body weight and symptoms. Half of them were euthanized at 4 days post-infection (dpi) to obtain the right lung and brain for viral titration and the left lung for histological staining (**Figure 4A**).

For SARS-CoV-2 Alpha infection, P36-5D2-treated animals maintained relatively stable body weights with 100% survival, whereas those without treatment suffered drastic loss of body weight starting from 5 dpi and reached humane endpoint at 7 dpi (**Figures 4B, C**). The viral titers found at 4 dpi for the untreated group were on average 3.3 × 10^5^ ± 1.5 × 10^5^ PFU/tissue and brain 7.2 × 10^5^ ± 10^6^ PFU/tissue. In contrast, the P36-5D2-treated group showed minimum detectable levels of viruses in the lung and brain tissues (**Figures 4F, G**). To assess the extent of lung damage and pulmonary inflammation of the virus infection, we performed histopathological analysis on the lung sections using hematoxylin and eosin (H&E) staining. The untreated group infected by the SARS-CoV-2 Alpha variant revealed evidence of moderate to severe lung damage and inflammation with marked infiltration of inflammatory cells such as granulocytes. In contrast, in the P36-5D2 group, the lung tissue remained intact and well defined (**Figure 4J**).

For SARS-CoV-2 Beta infection, the untreated group exhibited progressive body weight loss from 3 to 5 dpi, and all succumbed to infection by 5 dpi. These results indicated that the disease progressed faster and with greater severity caused by SARS-CoV-2 Beta as compared to SARS-CoV-2 Alpha infection. Yet, P36-5D2 still conferred complete protection and the mice maintained stable body weights, except for one mouse experiencing a slight decline with mild symptoms at 6 dpi and recovering by 8 dpi (**Figures 4D, E**). In the untreated group, the viral titers found at 4 dpi were on average 789 ± 420 PFU/tissue and brain 7.0 × 10^4^ ± 10^5^ PFU/tissue. The prophylactic use of P36-5D2 against the SARS-CoV-2 Beta variant was able to reduce lung viral titers for 2 logs compared to that in the control animals. The brain titers of the P36-5D2 group were undetectable, except for one mouse (**Figures 4H, I**). Evaluation of H&E staining revealed that the lung sections from the untreated group infected by the Beta variant showed similar histopathological changes to those infected by the Alpha variant, with severe inflammatory infiltrates and edema. In contrast, in the P36-5D2 group, there was no damage or pulmonary inflammation in lung tissues (**Figure 4K**). These results strongly indicate the broad and potent neutralizing activity of P36-5D2 against the SARS-CoV-2 Alpha and Beta variants *in vivo via* prophylactic interventions.

## Discussion

We report here the structural and functional characterization of the human neutralizing antibody P36-5D2 isolated from SARS-CoV-2 convalescent individuals. Compared to many other isolated human mAbs, the most outstanding and unique feature of P36-5D2 is its breadth and potency against the SARS-CoV-2 Alpha, Beta, and Gamma variants, as well as mutant viruses derived from the variants. Passive delivery of P36-5D2 protected K18-hACE2 mice from infection with the SARS-CoV-2 Alpha or Beta variant and significantly reduced the viral loads in both the lungs and brain. Crystal and cryo-EM structural analyses showed that P36-5D2 recognized a highly conserved epitope on RBD, which is accessible in both “up” and “down” conformations and able to withstand the mutated residues K417N, E484K, and N501Y that compromised many neutralizing mAbs, including some approved for EUA. Recently, Hastie and colleagues conducted a comprehensive study to map the landscape of neutralizing epitopes on the RBD of SARS-CoV-2 (44). A total of 186 RBD-directed antibodies with potential therapeutic use were categorized into seven core “communities” (RBD-1 to RBD-7) based on their distinct footprints and broad competition profiles. RBD-1, RBD-2, and RBD-3 antibodies targeted the receptor-binding surface, while RBD-4 and RBD-5 antibodies bound to the outer face of RBD. RBD-6 and RBD-7 antibodies, on the other hand, bound to the inner face of RBD. In this context, P36-5D2 would fall into the community of RBD-5 antibodies together with REGN10987 and S309. Consistent with what we have found here, RBD-5 antibodies showed broad resistance to the SARS-CoV-2 variants, suggesting that this community of antibodies binds to the conserved epitopes across all variants. Similar to REGN10987 and S309, P36-5D2 barely competed with ACE2 for binding to RBD, suggesting that their mechanism of neutralization may involve immunoglobulin G (IgG)-mediated spike crosslinking on virions, steric hindrance, or aggregation of virions (41). Nevertheless, these results indicated that we have identified a broad and potent neutralizing antibody capable of overcoming the major circulating SARS-CoV-2 variants. P36-5D2 and the epitope it recognized may serve as an important reference for the development of next-generation antibody therapies and vaccines against SARS-CoV-2 infection.

A couple of points need to be highlighted here. Firstly, despite the rapid emergence and spread of the SARS-CoV-2 variants, infected and convalescent individuals can generate and produce RBD-specific antibodies with impressive breadth and potency. The number and relative proportion of such antibodies appeared to be small, but they may contribute substantially to the remaining serum neutralizing activity to the variants in recovered or vaccinated individuals, particularly after boosting with mRNA vaccines (24, 45, 46). Comprehensive characterization of this class of antibodies will provide us with deeper insights into their ontogeny and potential ways of inducing broader and more effective protection against the circulating and future variants. Secondly, although many of the current SARS-CoV-2 variants bear mutations in the RBD, the identification of P36-5D2 has revealed the existence of a highly conserved sequence and structure on RBD that could potentially be used to trigger more broad and potent immune responses, avoiding the current prevalent mutations in dampening the immune responses generated during natural infection and vaccination. This would require preferentially exposing the conserved regions on RBD while minimizing the receptor-binding motif that is commonly recognized during infection and vaccination. How to achieve this design and, more importantly, to realize the desired immune response will surely be a big challenge. However, recent advances in structure-based vaccine design through understanding of the antigen–antibody interaction will certainly offer new possibilities for this highly anticipated outcome. The identification of P36-5D2, as well as other similar mAbs (19, 20, 36–39), represents the first but important step for us to achieve the ultimate goal of developing a universal vaccine against all SARS-CoV-2 variants and beyond.

## Materials and Methods

### Study Approval

This study received approval from the Research Ethics Committee of Beijing Youan Hospital, China (LL-2020-039-K) and Shenzhen Third People’s Hospital (2020-084). The research was conducted in strict accordance with the rules and regulations of the Chinese Government for the protection of human subjects. The study subjects agreed and signed the written informed consent for the use of their blood samples in research. All animal experiments were conducted in a Biosafety Level 3 (BSL-3) facility in accordance with the National University of Singapore (NUS) Institutional Animal Care and Use Committee (IACUC) (protocol no. R20-0504), and the NUS Institutional Biosafety Committee (IBC) and NUS Medicine BSL-3 Biosafety Committee (BBC) approved SOPs.

### Cell Lines

HEK293T cells (ATCC, Manassas, VA, USA), HeLa cells expressing hACE2 orthologs (kindly provided by Dr. Qiang Ding), and Vero E6 cells (ATCC) were maintained at 37°C in 5% CO_2_ in Dulbecco’s minimal essential medium (DMEM) containing 10% (*v*/*v*) heat-inactivated fetal bovine serum (FBS) and 100 U/ml penicillin–streptomycin. FreeStyle 293F cells (R79007; Thermo Fisher Scientific, Waltham, MA, USA) were maintained at 37°C in 5% CO_2_. Sf9 cells and Hi5 cells (ATCC) were maintained at 27°C in Sf-900 II SFM medium.

### Recombinant Protein Expression and Purification

Recombinant SARS-CoV-2 RBD (residues R319–F541) and trimeric spike for SARS-CoV-2 (residues M1–Q1208) and the N-terminal peptidase domain of human ACE2 (residues S19–D615) were expressed using the Bac-to-Bac Baculovirus Expression System (Invitrogen, Carlsbad, CA, USA) as previously described (9, 47). The SARS-CoV-2 spike contains proline substitutes at residues 986 and 987, “GSAS” substituted at the furin cleavage site (residues 682–685), and C-terminal foldon trimerization motif, Strep-tag, and six-histidine tag. The SARS-CoV-2 RBD, RBD-3M, carrying K417N/E484K/N501Y mutations and ACE2 peptidase domain contains a C-terminal six-histidine tag. These recombinant protein genes were cloned into pFastBac-Dual vectors (Invitrogen) and transformed into DH10Bac component cells. The bacmid was extracted and transfected into Sf9 cells using Cellfectin II Reagent (Invitrogen). The recombinant baculoviruses were extracted from the transfected supernatant and amplified to generate high-titer virus stock. Viruses were then used to infect Hi5 cells for recombinant protein expression.

### Antibody and Fab Production

Antibody and Fab production was conducted as previously described (47, 48). The published SARS-CoV-2 mAbs REGN10933, CB6, BD368-2, REGN10987, and 4A8 and the negative control antibody 2G4 against Ebola virus (EBOV) glycoprotein (GP) were synthesized according to the sequences released in Protein Data Bank (PDB) (26, 27, 32, 40, 49). Antibodies were produced by transient transfection of HEK 293F cells (Life Technologies, Carlsbad, CA, USA) using equal amounts of paired heavy- and light-chain plasmids by PEI (Sigma, St. Louis, MO, USA). After 5 days, the culture supernatant containing antibodies was collected and purified with protein A microbeads (GenScript, Piscataway, NJ, USA) according to the manufacturer’s protocol. Beads were collected using the magnetic separation rack and the antibodies eluted from beads with an elution buffer (0.3 M glycine, pH 2.0) into a neutralization buffer (1 M Tris-HCl, pH 8.0), followed by dialyzing into phosphate-buffered saline (PBS). Concentrations were determined by BCA Protein Assay kits (Thermo Scientific, Waltham, MA, USA). To produce Fab fragments, the antibodies were cleaved using Protease Lys-C (Sigma) with an IgG/Lys-C ratio of 4000:1 (*w*/*w*) in 10 mM EDTA, 100 mM Tris-HCl, pH 8.5, at 37°C for 12 h. Fc fragments were removed using Protein A microbeads.

### Isolation of Spike-Specific Single B Cells by FACS

Spike-specific single B cells were sorted as previously described (28). Peripheral blood mononuclear cells (PBMCs) from individuals infected with SARS-CoV-2 were collected and incubated with 500 nM SARS-CoV-2 spike for 30 min at 4°C, followed by antibody cocktail for the identification of spike-specific B cells. The cocktail consisted of CD3-PE-Cy5 (BD Biosciences, Franklin Lakes, NJ, USA) at a 1:25 dilution, CD14-PE-Cy5 (eBioscience, San Diego, CA, USA) at a 1:50 dilution, CD16-PE-Cy5 (BD Biosciences) at a 1:25 dilution, CD235a-PE-Cy5 (BD Biosciences) at a 1:100 dilution, CD20-PE-Cy7 (BD Biosciences) at a 1:200 dilution, CD27-BV421 (BD Biosciences) at a 1:50 dilution, IgG–fluorescein isothiocyanate (FITC) (BD Biosciences) at a 1:25 dilution, anti-His-APC (BioLegend, San Diego, CA, USA) at a 1:20 dilution, and streptavidin–phycoerythrin (PE) (eBioscience) at a 1:100 dilution. The stained cells were washed with FACS buffer (PBS containing 2% FBS) and resuspended in 500 μl FACS buffer before being stained with propidium iodide (PI) (eBioscience). Spike-specific single B cells were gated as live^+^CD3^−^CD14^−^CD16^−^CD235a^−^CD20^+^CD27^+^IgG^+^Spike^+^ and sorted into 96-well PCR plates containing 2 μl of lysis buffer (1.9 μl 0.2% Triton X-100, 0.1 μl RNase inhibitor; Clontech, Mountain View, CA, USA) per well. The plates were then snap-frozen on dry ice and stored at −80°C until the reverse transcription reaction.

### Single B-Cell PCR and Construction of Antibody Genes

The IgG heavy- and light-chain variable genes were amplified by nested PCR and cloned into linear expression cassettes or expression vectors to produce full IgG1 antibodies as previously described (28). Specifically, all second-round PCR primers containing tag sequences were used to produce the linear Ig expression cassettes by overlapping PCR. Separate primer pairs containing the specific restriction enzyme cutting sites (heavy chain, 5′-*Age*I/3′-*Sal*I; kappa chain, 5′-*Age*I/3′-*Bsi*WI; and lambda chain, 5′-*Age*I/3′-*Xho*I) were used to amplify the cloned PCR products. The PCR products were purified and cloned into the backbone of the antibody expression vectors containing the constant regions of human IgG1. Overlapping PCR products of paired heavy- and light-chain expression cassettes were co-transfected into HEK 293F cells.

### Neutralization Activity of mAbs Against Pseudotyped SARS-CoV-2

WT and mutated SARS-CoV-2 pseudoviruses were generated by co-transfection of human immunodeficiency virus backbones expressing firefly luciferase (pNL43R-E-luciferase) and pcDNA3.1 (Invitrogen) expression vectors encoding the respective spike proteins into 293T cells (ATCC) (20). Viral supernatants were collected 48 h later. Viral titers were measured as the luciferase activity in relative light units (Bright-Glo Luciferase Assay Vector System; Promega Biosciences, WI, USA). Serial dilutions of mAbs were prepared with the highest concentration of 5 or 50 μg/ml. WT or mutated spike pseudoviruses were mixed with mAbs and incubated at 37°C for 1 h. HeLa-hACE2 cells (1.5 × 10^4^ per well) were then added into the mixture and incubated at 37°C for 60 h before cell lysis for measuring the luciferase activity. The percent of neutralization was determined by comparing with the virus control.

### Neutralization Activity of mAbs Against Infectious SARS-CoV-2

A plaque reduction neutralization test against infectious SARS-CoV-2 WT, shown in **Supplementary Figure S1A**, was performed in a Chinese certified BSL-3 laboratory. Neutralization assays against infectious SARS-CoV-2 WT were conducted using a clinical isolate (Beta/Shenzhen/SZTH-003/2020, EPI_ISL_406594 at GISAID) previously obtained from a nasopharyngeal swab of an infected patient (28). Serial dilutions of the test antibodies were conducted, mixed with 75 μl of SARS-CoV-2 [8 × 10^3^ focus-forming units (FFU)/ml] in 96-well Microwell plates, and incubated for 1 h at 37°C. The mixtures were then transferred into 96-well plates, seeded with Vero E6 cells, and allowed absorption for 1 h at 37°C. The inocula were then removed before adding the overlay media (100 μl MEM containing 1.6% carboxymethyl cellulose). The plates were then incubated at 37°C, 5% CO_2_ for 24 h. Cells were fixed in 4% formaldehyde solution for 30 min and the overlays removed. Cells were permeabilized with 0.2% Triton X-100 and incubated with cross-reactive rabbit anti-SARS-CoV-N IgG (Sino Biological, Beijing, China) for 1 h at room temperature before adding horseradish peroxidase (HRP)-conjugated goat anti-rabbit IgG (H+L) antibody (Jackson ImmunoResearch, West Grove, PA, USA). Cells were further incubated at room temperature. The reactions were developed with KPL TrueBlue Peroxidase substrates (Seracare Life Sciences, Milford, MA, USA). The numbers of SARS-CoV-2 foci were calculated using an EliSpot reader (Cellular Technology, Shaker Heights, OH, USA).

The plaque assay against infectious SARS-CoV-2 VOCs, shown in **Figure 1C**, was performed in the National University of Singapore certified BSL-3 laboratory. The infectious SARS-CoV-2 used in the neutralization belongs to GISAID lineage clade L (WT, B) (accession ID: EPI_ISL_574502), GRY (Alpha, B.1.1.7) (accession ID: EPI_ISL_754083), and GH (Beta, B.1.351.3) (accession ID: EPI_ISL_1173248). Serial dilutions of the test antibodies were conducted, mixed with 50 PFU infectious SARS-CoV-2 in 12-well plates, and incubated for 1 h at 37°C. The mixtures were then transferred into 12-well plates, seeded with Vero E6 cells, and allowed absorption for 1 h at 37°C. The inocula were then removed and the wells washed once with PBS before adding the overlay media [1 ml DMEM containing 1.2% microcrystalline cellulose (MCC)]. The plates were then incubated at 37°C, 5% CO_2_ for 72 h for plaque formation. Cells were fixed in 10% formalin overnight before counterstaining with crystal violet. The virus titer of each dilution was determined through the number of plaques formed and expressed in neutralization percentage in comparison to positive control.

### Antibody Binding Kinetics, Competition with Receptor ACE2, Epitope Mapping Measured by SPR

The binding kinetics of mAbs to SARS-CoV-2 RBD were analyzed using SPR (Biacore 8K; GE Healthcare, Chicago, IL, USA). Specifically, recombinant protein A (Sino Biological) or the anti-His antibody (Cytiva, Marlborough, MA, USA) was covalently immobilized to a CM5 sensor chip *via* amine groups in 10 mM sodium acetate buffer (pH 4.5) for a final RU (response units) around 7,000. The running buffer HBS-EP was composed of 0.01 M HEPES, pH 7.4, 0.15 M NaCl, and 0.05% (*v*/*v*) Tween-20. The IgG form of mAbs was captured by the sensor chip immobilized with recombinant protein A, and then serial dilutions of SARS-CoV-2 RBD flowed through the sensor chip system. The resulting data were fitted to a 1:1 binding model using the Biacore 8K Evaluation software (GE Healthcare).

To determine competition with the human ACE2, the SARS-CoV-2 RBD was immobilized to a CM5 sensor chip *via* the amine group for a final RU around 250. In the first round, antibodies (1 μM) were injected onto the chip for 120 s to reach binding steady state and HBS-EP was injected for 120 s. In the second round, antibodies (1 μM) were injected onto the chip for 120 s and ACE2 (2 μM) was then injected for 120 s. In the third round, HBS-EP was injected for 120 s and ACE2 (2 μM) was then injected for 120 s. The sensorgrams of the three rounds were aligned from 120 to 240 s in Biacore 8K Evaluation software (GE Healthcare). The blocking efficacy was determined by a comparison of the response units with and without prior antibody injection.

For epitope mapping, the SARS-CoV-2 RBD was immobilized to a CM5 sensor chip *via* the amine group for a final RU around 250. Two different mAbs were sequentially injected and monitored for binding activity to determine whether these two mAbs recognized separate or closely situated epitopes.

### Binding of mAbs to Cell Surface-Expressed WT and Mutated Spikes

The entire procedure was conducted as previously described (20). HEK 293T cells were transfected with expression plasmids encoding either WT or mutated full-length spike of SARS-CoV-2, and incubated at 37°C for 36 h. Cells were removed from the plate using trypsin and distributed into 96-well plates for individual staining. Cells were washed twice with 200 μl staining buffer (PBS with 2% FBS) between each of the following. Firstly, cells were stained with 2 μg/ml testing mAb and 5 μg/ml S2-specific monoclonal antibody (MP Biomedicals, Santa Ana, CA, USA) at 4°C for 30 min in 100 μl staining buffer. Then, PE-labeled anti-human IgG Fc (BioLegend) at a 1:100 dilution and anti-mouse IgG FITC (Thermo Fisher Scientific) at a 1:200 dilution were added into 40 μl staining buffer at room temperature for 30 min. After extensive washes, the cells were resuspended and analyzed with BD LSRFortassa (BD Biosciences) and FlowJo v10 software (FlowJo). HEK 293T cells without transfection were also stained as a background control. The RBD-specific mAbs REGN10933, CB6, BD368-2, and REGN10987 were used as positive control mAbs, while 2G4 was used as a negative control mAb. HEK 293T cells with mock transfection were stained as a background control.

### Antibody Protection in hACE2 Transgenic Mice

Eight-week-old female K18-hACE2 transgenic mice (InVivos Ptd Ltd, Lim Chu Kang, Singapore) were used for this study. The mice were housed and acclimatized in an ABSL-3 facility for 72 h prior to the start of the experiment.

K18-hACE2 transgenic mice were subjected to pretreatment of mAb P36-5D2 (10 µg/kg) delivered through IP injection a day prior to infection. The inoculation of mice was conducted through IN delivery with 25 µl, 10^3^ PFU of the infectious SARS-CoV-2 Alpha or Beta variant. Baseline body weights were measured prior to infection and monitored daily by two personnel post-infection for the duration of the experiment. To assess the viral load, mice from each experimental group were sacrificed 4 dpi, with brain and lung tissues harvested. Each organ was halved for the plaque assay and histology, respectively. Tissues were homogenized with 0.5 ml DMEM supplemented with antibiotic and antimycotic (Gibco, Waltham, MA, USA) and titrated in Vero E6 cells using plaque assays.

For virus titer determination, supernatants from homogenized tissues were diluted 10-fold serially in DMEM supplemented with antibiotic and antimycotic. Of each serial diluted supernatant, 250 µl was added to Vero E6 cells into 12-well plates. After 1 h of incubation for virus adsorption, the inoculum was removed and washed once with PBS. About 1.2% MCC-DMEM supplemented with antibiotic and antimycotic overlay media was added to each well and incubated at 37°C, 5% CO_2_ for 72 h for plaque formation. The cells were then fixed in 10% formalin overnight and counterstained with crystal violet. The number of plaques was determined and the virus titers of individual samples were expressed in logarithm of PFU per organ.

### Histopathological Analyses

Left lung lobes were fixed in 3.7% formaldehyde solution prior to removal from BSL-3 containment. The tissues were routinely processed, embedded in paraffin blocks (Leica Surgipath Paraplast), sectioned at 5-µm thickness, and stained with H&E (Thermo Scientific) following standard histological procedures. The extent and severity of lung damage was qualitatively described as no infection, mild, moderate, or severe.

### Crystal Analysis and Structural Determination

The antibody P36-5D2 Fab and RBD-3M were mixed at a molar ratio of 1:1.5 and incubated at 4°C for 60 min. The mixture was purified by gel filtration pre-equilibrated by 1× HBS buffer. The complex of P36-5D2 Fab and RBD-3M was concentrated to 11 mg/ml for crystallization. Crystal of the P36-5D2 Fab and RBD-3M complex was obtained after 5 days using the sitting drop method. The well solution was 0.1 M sodium HEPES 7.5, 10% (*w*/*v*) PEG 6000, and 5% (*v*/*v*) MPD. Diffraction data were collected at SSRFBL18U1 beam line of the Shanghai Synchrotron Research Facility (SSRF). The data were processed by HKL3000 and the structure was determined using the molecular replacement method with PHASER in the CCP4 suite (50). Model building and refinement were performed using COOT v.0.9.2 and PHENIX v.1.18.2, respectively (51, 52). The data processing statistics are listed in **Supplementary Table S1**.

### Cryo-EM Sample Preparation and Data Collection

Aliquots of complex of SARS-CoV-2 spike ectodomains and P36-5D2 Fab (4 μl, 1.6 mg/ml, in buffer containing 20 mM Tris, pH 8.0, and 150 mM NaCl) were applied to glow-discharged holey carbon grids (Quantifoil grid, Au 300 mesh, R1.2/1.3). The grids were then blotted for 2 s and plunge-frozen into liquid ethane using Vitrobot Mark IV (Thermo Fisher Scientific).

Images for complex were recorded using the FEI Titan Krios microscope (Thermo Fisher Scientific) operating at 300 kV with a Gatan K3 Summit direct electron detector (Gatan Inc., Pleasanton, CA, USA) at Tsinghua University. The automated software AutoEMation2 (53) was used to collect 4,534 movies for complex of SARS-CoV-2 spike ectodomains and P36-5D2 Fab in super-resolution mode at a nominal magnification of ×29,000 and at a defocus range between −1.5 and −1.8 μm. Each movie has a total accumulated exposure of 50 e-/Å^2^ fractionated in 32 frames of 2.13-s exposures. The final image was binned twofold to a pixel size of 0.97 Å. The data collection statistics are summarized in **Supplementary Table S3**.

### Cryo-EM Data Processing

Motion correction (MotionCor2 v.1.2.6) (54), CTF estimation (GCTF v.1.18) (55), and non-templated particle picking (Gautomatch v.0.56; http://www.mrc-lmb.cam.ac.uk/kzhang/) were automatically executed using the TsingTitan.py program. Sequential data processing was carried out on RELION-3.1 (56). Initially, ~910,000 particles were subjected to 2D classification. After three additional 2D classifications, the best selected 480,469 particles were applied for the initial model and 3D classification. A subset of 242,871 particle images from state 1 (one RBD up and two RBDs down) and 225,216 particle images from state 2 (two RBDs up and one RBD down) were further subjected to 3D auto-refine and post-processing. The final resolutions for states 1 and 2 were 3.69 and 3.65 Å, respectively. The interface between the S protein of SARS-CoV-2 and Fab was subjected to focused refinement with mask on the region of the RBD–Fab complex to improve the map quality. The selected 480,469 particles were 3D classified focused on the RBD–Fab complex. Then, the good particles were selected for focused refinement and post-processing with a final resolution of 3.8 Å. The resolution was estimated with the gold-standard Fourier shell correlation 0.143 criterion. Details of the data collection and processing are shown in **Supplementary Figure S4** and **Table S3**.

### Cryo-EM Model Building and Refinement

The initial model of complex of SARS-CoV-2 spike ectodomains and P36-5D2 Fab was generated using the models (PDB 7A94 and 7A97) and fit into the map using UCSF Chimera v.1.15 (57). Manual model rebuilding was carried out using COOT v.0.9.2 (51) and refined with PHENIX v.1.18.2 (52) real-space refinement. The quality of the final model was analyzed with PHENIX v.1.18.2 (52). The validation statistics of the structural models are summarized in **Supplementary Table S3**. All structural figures were generated using PyMOL 2.0 (58) and Chimera v.1.15 (57).

### Quantification and Statistical Analysis

The technical and independent experiment replicates were indicated in the figure legends. The half-maximal inhibitory concentration (IC_50_) and 90% inhibitory concentration (IC_90_) of the mAbs were calculated using the four-parameter dose inhibition equation in Graphpad Prism 9.0. Percentages of the binding of mAbs to cell surface-expressed SARS-CoV-2 variants were calculated as the ratio between the mutated over WT mean fluorescence intensity (MFI) normalized relative to that of the S2-specific antibody. All MFI values were weighted by multiplying the number of positive cells in the selected gates. The fold change of the mutant spike relative to WT D614G in binding or neutralization was calculated by simple division of the respective IC_50_ or MFI values. In animal experiments, a two-tailed unpaired *t*-test was used to assess statistical significance. Statistical calculations were performed in GraphPad Prism 9.0. Differences with *p*-values less than 0.05 were considered to be statistically significant (*p < 0.05, **p < 0.01; n.s., not significant).

## Data Availability Statement

The datasets presented in this study can be found in online repositories. The names of the repository/repositories and accession number(s) can be found in the article/**Supplementary Material**.

## Ethics Statement

The studies involving human participants were reviewed and approved by the Research Ethics Committee of Beijing Youan Hospital, China (LL-2020-039-K) and Shenzhen Third People’s Hospital (2020-084). The patients/participants provided written informed consent to participate in this study. The animal study was reviewed and approved by the National University of Singapore (NUS) Institutional Animal Care and Use Committee (IACUC) and the NUS Institutional Biosafety Committee (IBC) and NUS Medicine BSL-3 Biosafety Committee (BBC).

## Author Contributions

LZ, JC, XW, and JW conceived, designed, and supervised the entire study. SS and JLi did antibody isolation and sequencing under the conduction of JW. SS and ZY did antibody binding analysis and pseudovirus neutralization. CM, ZA, and JC performed the antibody protection experiment in K18-hACE2 mice. SZ did the cryo-EM structure of the Fab-spike complex. JLa collected the crystal structure of the Fab-RBD complex. CM, ZA, LC, and ZZ carried out the infectious virus neutralization assays. JY provided the SARS-CoV-2 spike protein. ZZ, TZ, XS, QZ, and RW conducted the antibody evaluation, structural analysis, and animal experiment. SS, CM, SZ, JLa, JLi, and LZ had full access to the data in the study, generated the figures and tables, and took responsibility for the integrity and accuracy of the data presentation. LZ, SS, CM, XW, and ZA wrote the original draft. LZ, JC, XW, and JW reviewed and edited the manuscript. All authors contributed to the article and approved the submitted version.

## Funding

This study was funded by the National Key Plan for Scientific Research and Development of China (2020YFC0848800 and 2020YFC0849900), the National Natural Science Foundation (81530065, 91442127, and 32000661), Beijing Municipal Science and Technology Commission (D171100000517 and Z201100005420019), the Science and Technology Innovation Committee of Shenzhen Municipality (202002073000002), COVID-19 Science and Technology Project of Beijing Hospitals Authority (YGZX-C1), Beijing Advanced Innovation Center for Structural Biology, Tsinghua University Scientific Research Program (20201080053 and 2020Z99CFG004), Tencent Foundation, Shuidi Foundation, TH Capital, and the National Science Fund for Distinguished Young Scholars (82025022), Singapore National Medical Research Council Centre Grant Program (CGAug16M009).

## Conflict of Interest

The authors declare that the research was conducted in the absence of any commercial or financial relationships that could be construed as a potential conflict of interest.

## Publisher’s Note

All claims expressed in this article are solely those of the authors and do not necessarily represent those of their affiliated organizations, or those of the publisher, the editors and the reviewers. Any product that may be evaluated in this article, or claim that may be made by its manufacturer, is not guaranteed or endorsed by the publisher.

## Supplementary Material

Supplementary Figure 1 SARS-CoV-2 spike-specific neutralizing antibodies screening and evaluation. **(A)** Antibody neutralization against pseudotyped and infectious SARS-CoV-2. Antibodies included eight isolated neutralizing mAbs P33-1F1, P33-3C5, P36-1A3, P36-1B7, P36-1C5, P36-5D2, P36-8F2 and P74-6D2 from SARS-CoV-2 infected individuals, four RBD-specific representative mAbs REGN10933, CB6, BD368-2, REGN10987, one NTD-specific representative mAb 4A8 and one negative control mAb 2G4. Data are presented as the means ± SEM from three independent experiments. **(B)** Binding kinetics of the top four isolated neutralizing mAbs P36-1A3, P36-1B7, P36-5D2 and P74-6D2 with SARS-CoV-2 RBD measured by SPR. The black lines indicate the experimentally derived curves while the red lines represent fitted curves based on the experimental data. Results presented are representatives of two independent experiments. **(C)** Antibody competition with ACE2 for binding to SARS-CoV-2 RBD measured by SPR. The plots show distinct binding patterns of ACE2 to the RBD with (colored curve) or without (black curve) prior incubation with each tested mAb. The results are representative of two independent experiments and color-coded for each mAb. **(D)** Epitope mapping through competitive binding measured by SPR. Pairs of antibodies were sequentially applied to SARS-CoV-2 RBD immobilized sensor chip. The level of reduction in response unit comparing with or without prior antibody incubation is the criterion for determining the two antibodies recognize the separate or closely situated epitopes. **(E)** Summary of antibody competition in **(D)**, in which ‘+++’ indicates >70% competition; ‘++’ 50–70%; ‘+’ 20–50%; and ‘−’ <20%.

Supplementary Figure 2 Neutralization of SARS-CoV-2 variants by each antibody, related to Figure. 1. Pseudoviruses bearing the indicated mutations were tested against serial dilutions of each mAb. Neutralizing activity was defined as the percent reduction in luciferase activities compared to no antibody controls. Levels of resistance were calculated as the -fold change in IC_50_ between each mutant and WT D614G, as presented in Figure. 1A. Results are presented as the mean value from three independent experiments.

Supplementary Figure 3 Binding to cell surface expressed SARS-CoV-2 variants by each antibody, related to Figure. 1. Wildtype and mutated spike were expressed on the surface on HEK 293T, incubated with the mAbs, followed by staining with anti-human IgG Fc PE and analysed by FACS. The gated cell percentages are shown. The fold changes in antibody binding, as shown in Figure. 1B, was determined by comparing the total MFI in the selected gate between spike variants and WT D614G. S2 mAb is a positive control antibody used for spike expression normalization. 2G4 targeting EBOV GP is negative control antibody. NC is HEK 293T cells with mock transfection.

Supplementary Figure 4 Cryo-EM data processing workflow. **(A)** Processing workflow of the P36-5D and SARS-CoV-2 spike complex cryo-EM data. Local resolution map and particle orientation distribution of the P36-5D/spike complex with 2 “down” and 1 “up” RBD **(B)** or with 1 “down” and 2 “up” RBD **(C)**. **(D, E)** The corrected, unmasked, masked and phase randomised FSC for the cryo-EM reconstructions of the density maps of P36-5D2/spike complex with C3 symmetry. The final resolution of the P36-5D/spike complex with 2 “down” and 1 “up” RBD is 3.69 Å **(D)**. The final resolution of the P36-5D/spike complex with 1 “down” and 2 “up” RBD is 3.65 Å **(E)**.

Supplementary Table 1 Crystal structure data collection and refinement statistics.

Supplementary Table 2 Contacts between SARS-CoV-2 RBD-3M and P36-5D2 (distance cutoff 4 Å).

Supplementary Table 3 Cryo-EM data collection, refinement and validation statistics.
