# Supplementary figures and images for: A Potent and Protective Human Neutralizing Antibody Against SARS-CoV-2 Variants
Source: Front Immunol. 2021 Dec 13;12:766821. doi: 10.3389/fimmu.2021.766821 (PMC8710476; doi:10.3389/fimmu.2021.766821)

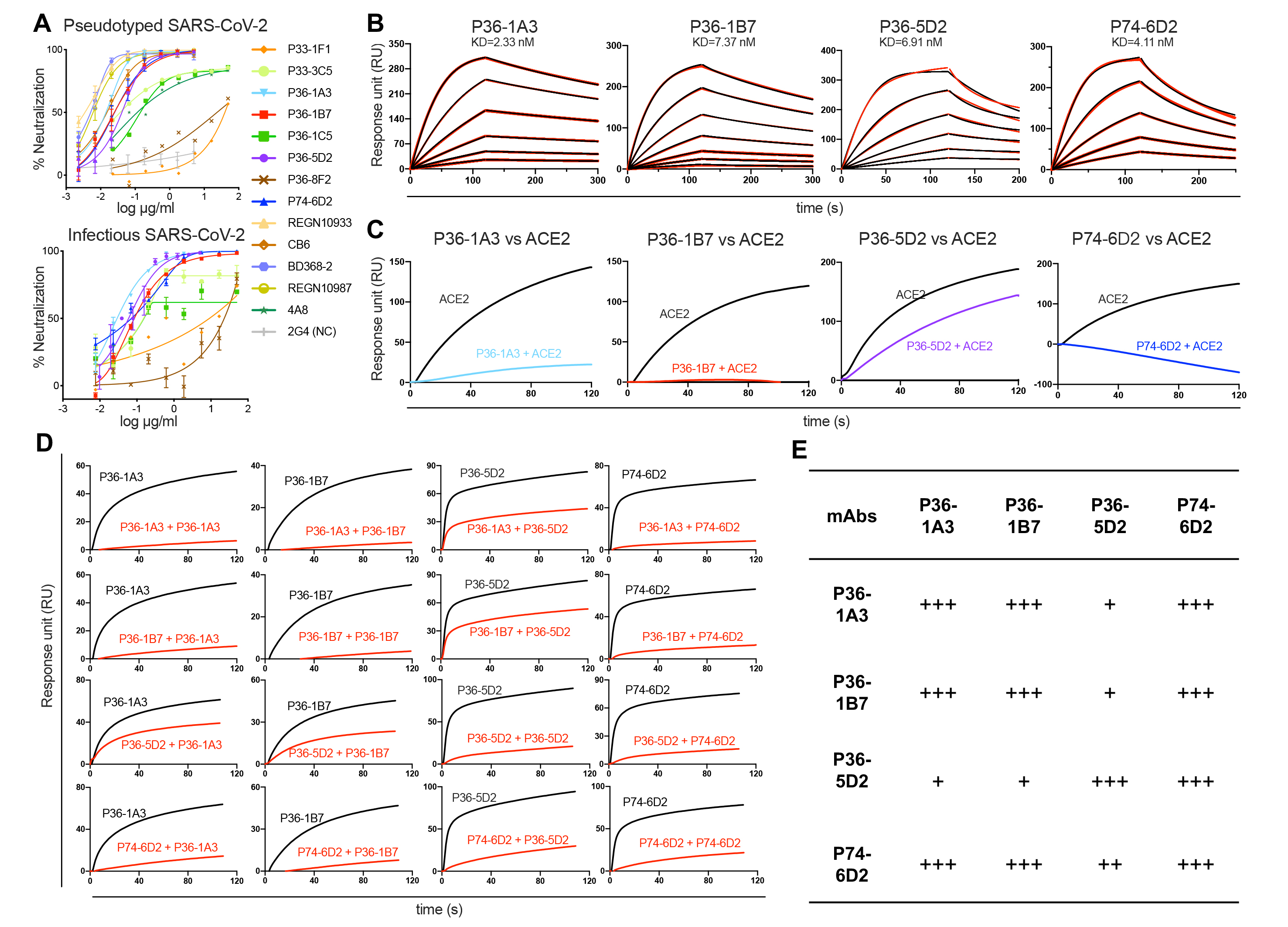

Supplement: Supplementary Figure 1 — SARS-CoV-2 spike-specific neutralizing antibodies screening and evaluation. (A) Antibody neutralization against pseudotyped and infectious SARS-CoV-2. Antibodies included eight isolated neutralizing mAbs P33-1F1, P33-3C5, P36-1A3, P36-1B7, P36-1C5, P36-5D2, P36-8F2 and P74-6D2 from SARS-CoV-2 infected individuals, four RBD-specific representative mAbs REGN10933, CB6, BD368-2, REGN10987, one NTD-specific representative mAb 4A8 and one negative control mAb 2G4. Data are presented as the means ± SEM from three independent experiments. (B) Binding kinetics of the top four isolated neutralizing mAbs P36-1A3, P36-1B7, P36-5D2 and P74-6D2 with SARS-CoV-2 RBD measured by SPR. The black lines indicate the experimentally derived curves while the red lines represent fitted curves based on the experimental data. Results presented are representatives of two independent experiments. (C) Antibody competition with ACE2 for binding to SARS-CoV-2 RBD measured by SPR. The plots show distinct binding patterns of ACE2 to the RBD with (colored curve) or without (black curve) prior incubation with each tested mAb. The results are representative of two independent experiments and color-coded for each mAb. (D) Epitope mapping through competitive binding measured by SPR. Pairs of antibodies were sequentially applied to SARS-CoV-2 RBD immobilized sensor chip. The level of reduction in response unit comparing with or without prior antibody incubation is the criterion for determining the two antibodies recognize the separate or closely situated epitopes. (E) Summary of antibody competition in (D), in which ‘+++’ indicates >70% competition; ‘++’ 50–70%; ‘+’ 20–50%; and ‘−’ <20%. [file Image_1.jpg]

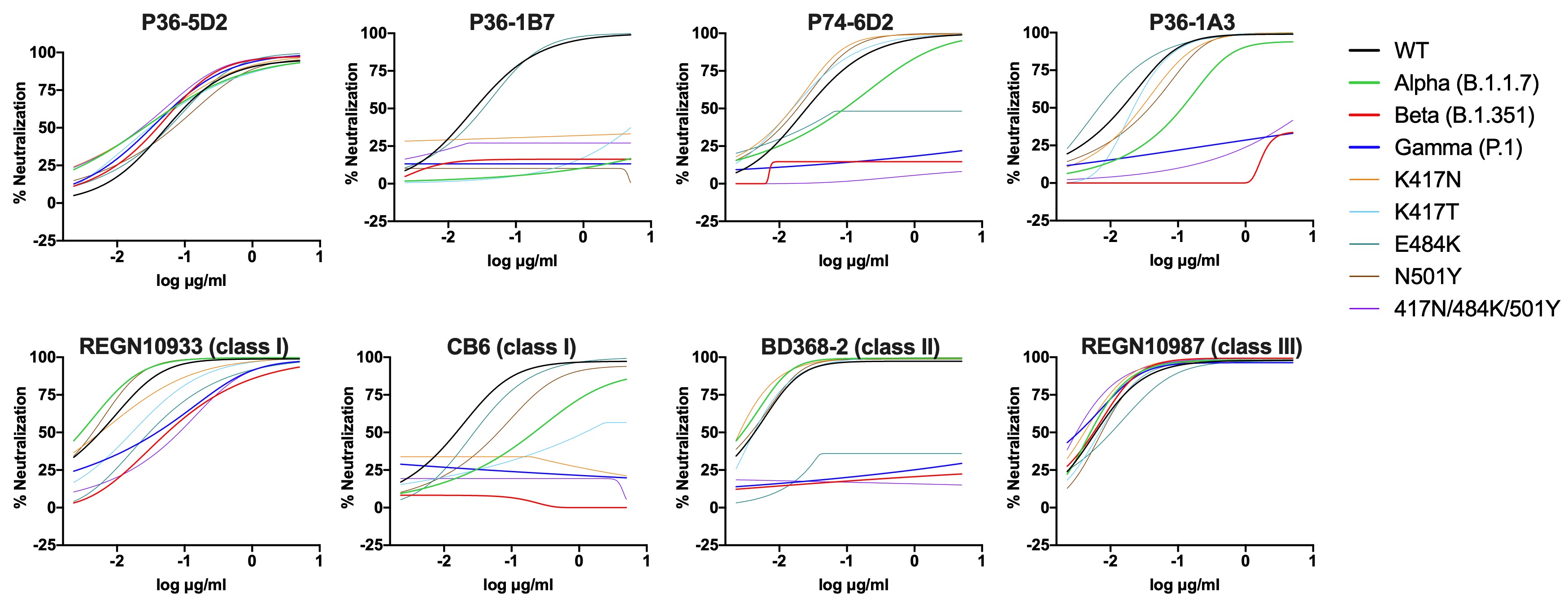

Supplement: Supplementary Figure 2 — Neutralization of SARS-CoV-2 variants by each antibody, related to Figure. 1. Pseudoviruses bearing the indicated mutations were tested against serial dilutions of each mAb. Neutralizing activity was defined as the percent reduction in luciferase activities compared to no antibody controls. Levels of resistance were calculated as the -fold change in IC50 between each mutant and WT D614G, as presented in Figure. 1A. Results are presented as the mean value from three independent experiments. [file Image_2.jpg]

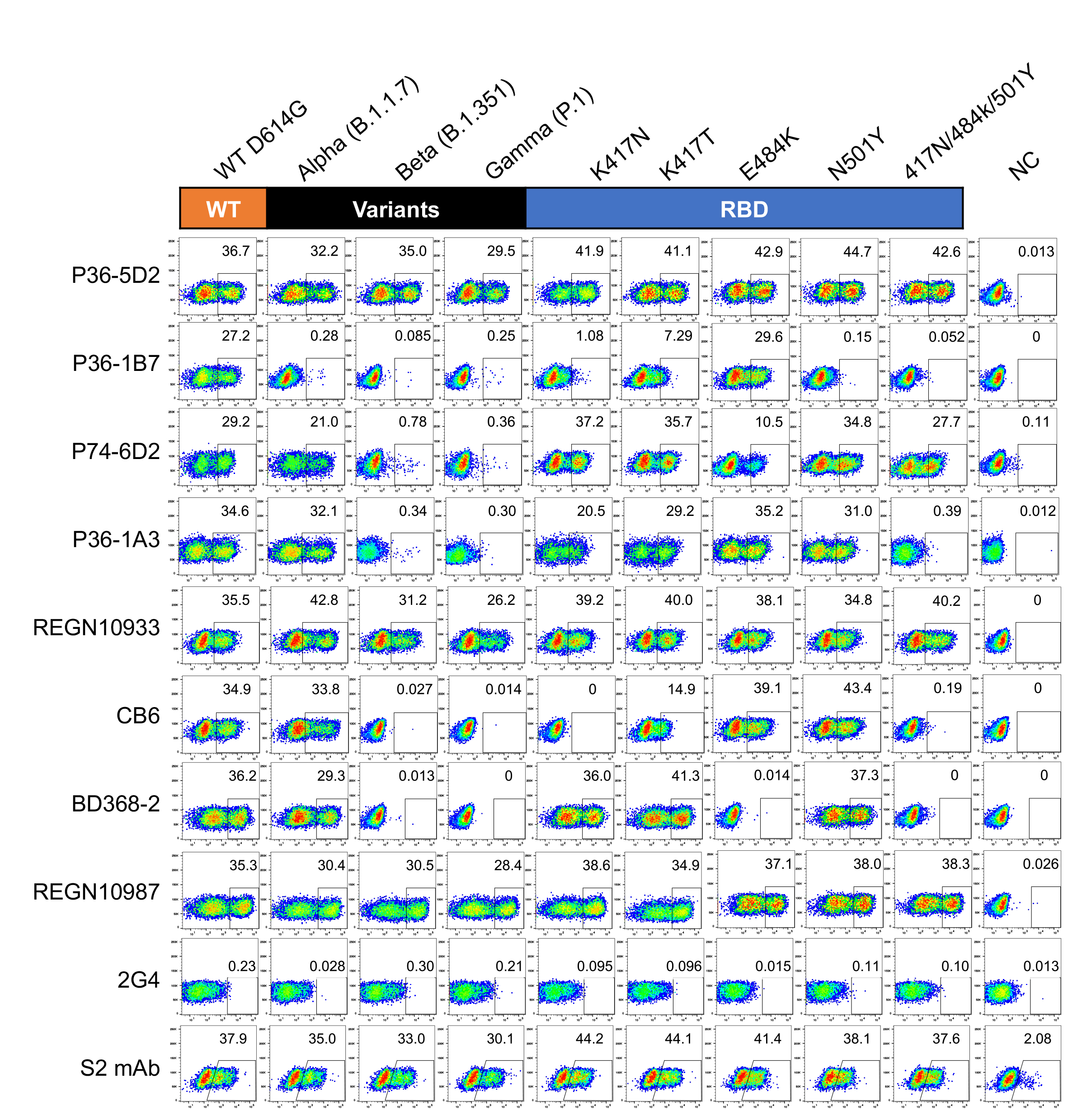

Supplement: Supplementary Figure 3 — Binding to cell surface expressed SARS-CoV-2 variants by each antibody, related to Figure. 1. Wildtype and mutated spike were expressed on the surface on HEK 293T, incubated with the mAbs, followed by staining with anti-human IgG Fc PE and analysed by FACS. The gated cell percentages are shown. The fold changes in antibody binding, as shown in Figure. 1B, was determined by comparing the total MFI in the selected gate between spike variants and WT D614G. S2 mAb is a positive control antibody used for spike expression normalization. 2G4 targeting EBOV GP is negative control antibody. NC is HEK 293T cells with mock transfection. [file Image_3.jpg]

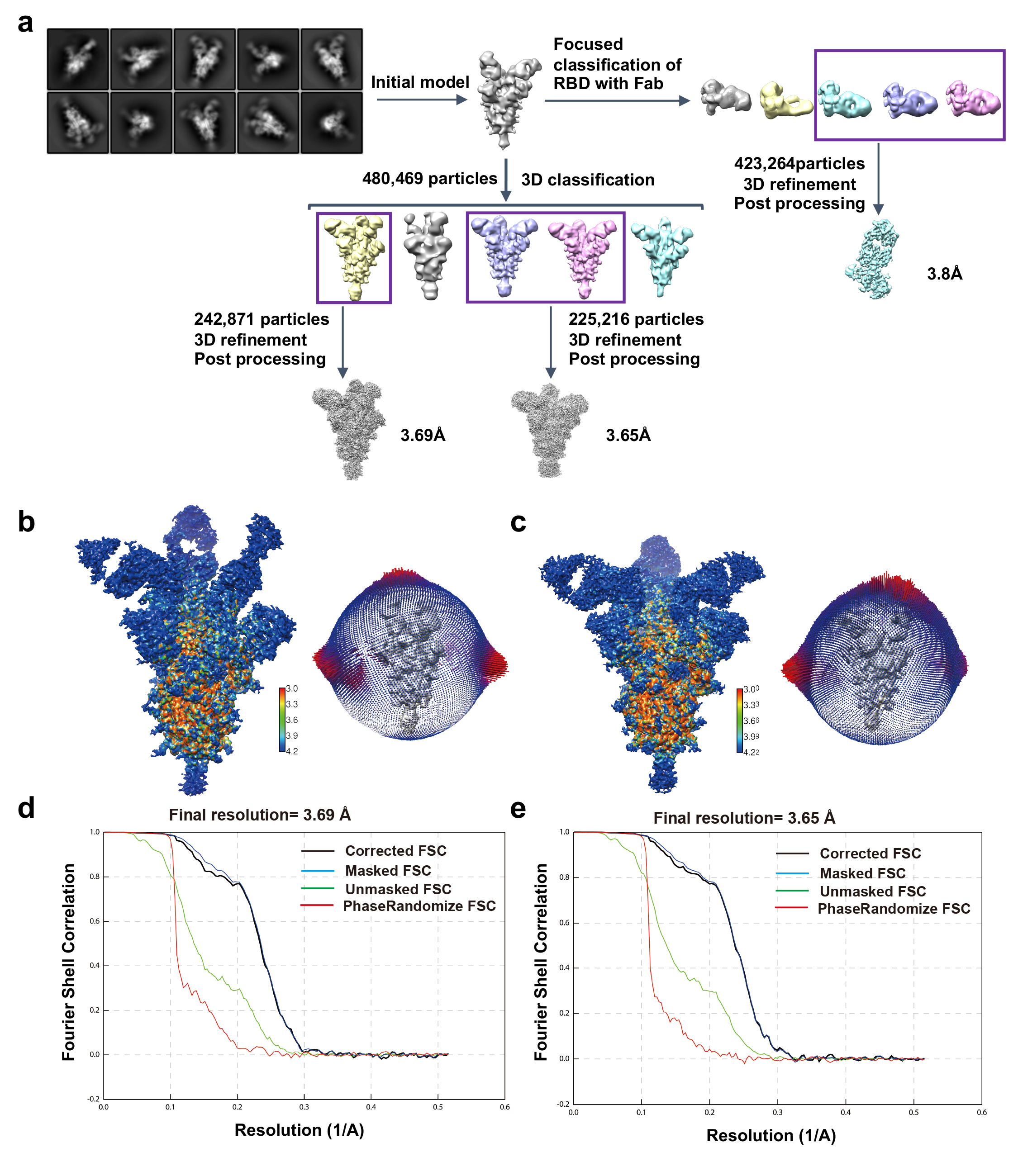

Supplement: Supplementary Figure 4 — Cryo-EM data processing workflow. (A) Processing workflow of the P36-5D and SARS-CoV-2 spike complex cryo-EM data. Local resolution map and particle orientation distribution of the P36-5D/spike complex with 2 “down” and 1 “up” RBD (B) or with 1 “down” and 2 “up” RBD (C). (D, E) The corrected, unmasked, masked and phase randomised FSC for the cryo-EM reconstructions of the density maps of P36-5D2/spike complex with C3 symmetry. The final resolution of the P36-5D/spike complex with 2 “down” and 1 “up” RBD is 3.69 Å (D). The final resolution of the P36-5D/spike complex with 1 “down” and 2 “up” RBD is 3.65 Å (E). [file Image_4.jpg]
